# Lymph node ratio, but not the total number of examined lymph nodes or lymph node metastasis, is a predictor of overall survival for pancreatic neuroendocrine neoplasms after surgical resection

**DOI:** 10.18632/oncotarget.19184

**Published:** 2017-07-12

**Authors:** Peng Liu, Xianbin Zhang, Yuru Shang, Lili Lu, Fei Cao, Min Sun, Zhaohui Tang, Brigitte Vollmar, Peng Gong

**Affiliations:** ^1^ Department of Hepatobiliary Surgery, The First Affiliated Hospital of Dalian Medical University, Dalian, 116011, China; ^2^ Institute of Experimental Surgery, University of Rostock, Schillingallee 69a, Rostock, 18059, Germany; ^3^ Department of Epidemiology, Dalian Medical University, Dalian, 116044, China; ^4^ Department of Oncology, Zhongnan Hospital of Wuhan University, Hubei Key Laboratory of Tumor Biological Behaviors and Hubei Cancer Clinical Study Center, Wuhan, 430071, China; ^5^ Department of General Surgery, Xinhua Hospital Shanghai Jiaotong University, Shanghai, 200092, China; ^6^ Dalian Key Laboratory of Hepatobiliary Pancreatic Diseases Prevention and Treatment and Liaoning Key Laboratory of Molecular Targeted Drugs in Hepatobiliary and Pancreatic Cancer, Dalian, 116011, China

**Keywords:** pancreatic neuroendocrine neoplasms, lymph node ratio, examined lymph nodes, lymph node metastasis, overall survival

## Abstract

**Aim:**

To evaluate the prognostic significance of lymph node metastasis, extent of examined lymph nodes (ELNs) and lymph node ratio (LNR) for resected pancreatic neuroendocrine neoplasms (pNENs).

**Materials and Methods:**

Surgically resected pNENs were assimilated from the Surveillance, Epidemiology, and End Results database. Kaplan-Meier and Cox proportional hazard models were used to examine the prognostic effect of clinicopathological characteristics on overall survival; Harrell’s concordance index was performed to assess the prognostic accuracy of all independent prognostic factors; and the Spearman’s rank correlation was used to assess the correlation between LNR and other clinicopathological characteristics.

**Results:**

Totally, 1,273 pathologically confirmed pNENs were included in our study. The extent of ELNs failed to show any survival benefit in entire cohort (ELNs ≤ 12 vs. ELNs > 12, *P* = 0.072) or pNENs without lymph node metastasis (ELNs ≤ 28 vs. ELNs > 28, *P* = 0.108). Lymph node metastasis and LNR > 0.40 were significantly (both *P* < 0.001) adverse prognostic factors of overall survival. However, only LNR > 0.40 was the independent predictor of survival after adjusted for other clinicopathological characteristics. Besides LNR, the age, gender, primary tumor site, grade and stage also were the independent predictors of overall survival; and this survival model had an acceptable predictive power (Harrell’s concordance index, 0.731).

**Conclusions:**

The current study suggested that the LNR, not the total number of ELNs and the lymph node metastasis, is an independent prognostic indicator of overall survival for pNENs after surgical resection.

## INTRODUCTION

Pancreatic neuroendocrine neoplasms (pNENs), also known as pancreatic endocrine tumors, islet cell neoplasms or islet cell carcinomas, are rare tumors with an annual incidence of 0.19/100,000–0.32/100,000 [1–3]. However, compared to 1973, the incidence of NENs in 2004 has increased 382% in United States [3]. Although pNENs are associated with relatively indolent physiological behavior, most patients eventually succumb to mortality due to the disease [4, 5].

Lymph node metastasis is commonly used as a critical prognostic factor for predicting survival and disease progression of pancreatic ductal adenocarcinomas (PDAC) and pNENs [6–9]. However, some studies showed that lymph node metastasis was not an independent prognostic factor of PDAC and pNENs [10, 11]. The accuracy of staging lymph node was directly proportional to the number of examined lymph nodes (ELNs), and many studies suggested that the extent of ELNs was significantly associated with survival of PDAC, especially in patients without lymph node metastasis [12–14]. Moreover, lymph node ratio (LNR), the number of metastatic lymph nodes divided by the total number of ELNs, was increasingly recognized as a more powerful prognostic factor than lymph node metastasis in PDAC [10, 11, 15], intraductal papillary mucinous [16], and ampullary carcinoma [17, 18].

However, the benefit of ELNs in pNENs is still unclear; and the role of LNR in predicting survival is contradictory. Boninsegna et al. [19] and Ricci et al. [20] proposed that LNR > 0.20 and LNR > 0.07, respectively, was the robust predictor of recurrence of pNENs. On the other hand, Murakami et al. [8] found that LNR > 0.20 did not correlate with poor overall survival (OS). These contradictory results might be attributed to the relative rarity of the samples and the cut-off values of LNR in these studies, which limited the identification of LNR in survival.

The Surveillance, Epidemiology, and End Results (SEER) database is an authoritative source of information with high-quality cancer registries, established in 1973, encompassing approximately 28% of the USA population. All the malignant cases were followed-up annually to determine the vital status. The large population and completed follow-up of SEER program can be safely speculated to represent the total USA population.

The aim of the present study was to use a large population to evaluate the predictive role of ELNs, lymph node metastasis, and LNR in OS of pNENs after surgical resection. Furthermore, we attempted to establish the correlation between LNR and clinicopathological characteristics (tumor size, grade, and stage).

## RESULTS

### Patients’ characteristics

**Figure 1:**
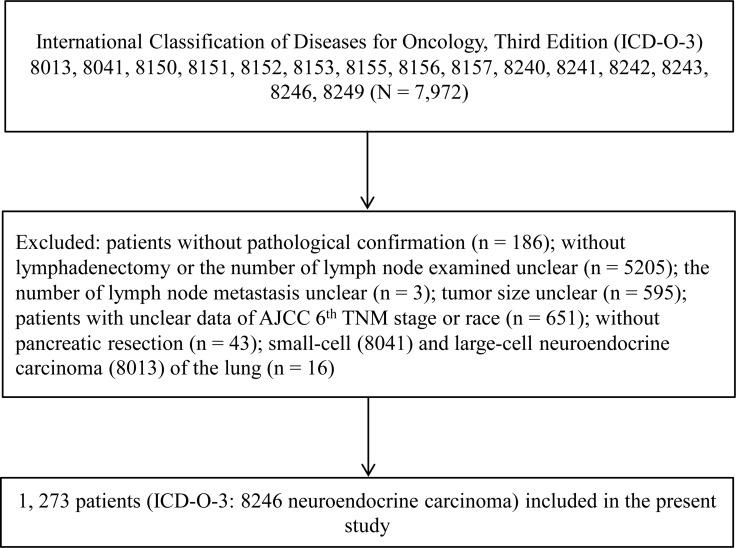
Flowchart of patients selection process

A total of 1,273 pathologically confirmed pNENs comprising of 680 males and 593 females, were included in our study (Figure 1). The median age and interquartile range (IQR) at diagnosis were 58 years (49 years–67 years). Approximately, 80% patients were ethnically Caucasians. The degree of tumor localized at the head was similar to that of the tail (32.8% and 36.8% of all patients, respectively, head-to-tail ratio, 0.89:1). Approximately, 60.0% of patients underwent partial pancreatectomy. The tumor size in 73.0% patients was larger than 2.0 cm. The median of ELNs was 10 (IQR, 5–16), and 42.0% patients with lymph node metastasis. The median of LNR was 0 (IQR, 0–0.2); 61.6% patients exhibited SEER grade I. The proportion of AJCC TNM stage I and stage II were 38.7% and 40.3%, respectively (Table 1).

**Table 1:** Clinicopathologic characteristics

	Patients (*N* = 1273)	
Characteristic	No.	%
Age		
≤ 60 years	732	57.50%
> 60 years	541	42.50%
Gender		
Male	680	53.42%
Female	593	46.58%
Race		
White	1022	80.28%
Black	131	10.29%
Others	120	9.43%
Prime Site		
Head	417	32.76%
Body	169	13.28%
Tail	469	36.84%
Others	218	17.12%
Surgical Procedures		
Enucleation	20	1.57%
Partial Pancreatectomy	736	57.82%
Total Pancreatectomy	136	10.68%
Whipple	348	27.34%
Surgery NOS	33	2.59%
Size		
≤ 2 cm	347	27.26%
> 2 cm	926	72.74%
Lymph Node		
Negative	742	58.29%
Positive	531	41.71%
SEER Grade		
I	784	61.59%
II	185	14.53%
III	95	7.46%
IV	16	1.26%
Unclear	193	15.16%
AJCC stage 6th		
I	493	38.73%
II	513	40.30%
III	24	1.89%
IV	243	19.09%

NOS, not otherwise specified; SEER Grade [34]; AJCC, American Joint Committee on Cancer.

### The lymph node metastasis and overall survival

**Figure 2:**
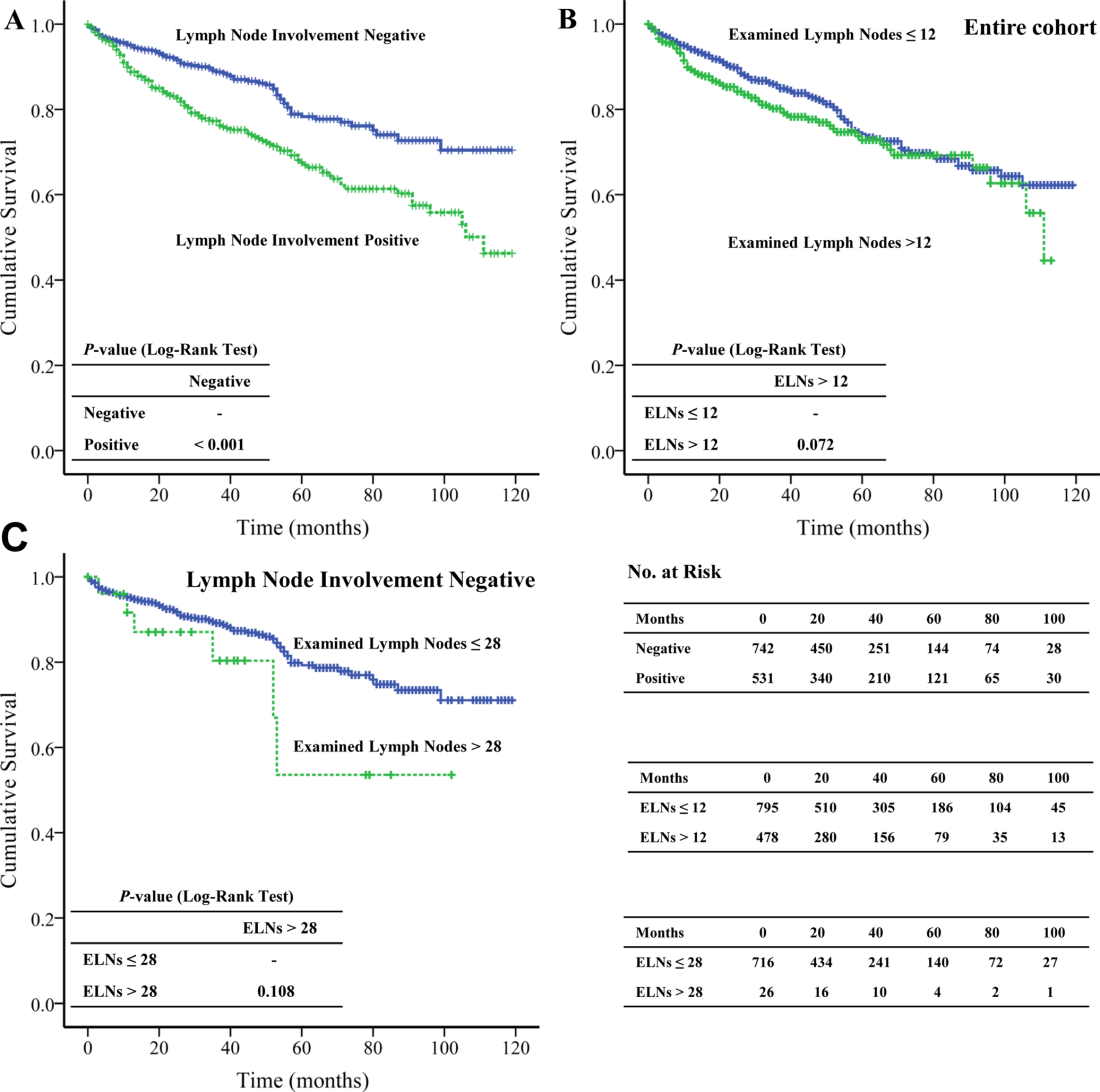
The Kaplan-Meier and log-rank tests of overall survival based on (**A**) lymph node metastasis; (**B**) the extent of examined lymph nodes of entire cohort; and (**C**) pNENs without lymph node metastasis.

As we assumed, the OS of the patients with lymph node metastasis (82.965 months ± 2.504 months) was significantly (*P* < 0.001) shorter than that of patients without metastasis (97.615 months ± 2.086 months) (Figure 2A). The univariate analysis showed the lymph node metastasis significantly (*P* < 0.001) increased the risk of death (HR 1.914, 95% CI: 1.467–2.497). However, the multivariate analysis failed to display that the lymph node metastasis was associated with OS (Table 2).

**Table 2:** Univariate and multivariate Cox regression analysis (LNR cut-off value, 0 and 0.40)

Variable	*N*	Univariate			Multivariate		
		*P*-value*	HR	95%CI	*P*-value	HR	95%CI
**Age**							
≤ 60 years	732	reference			reference		
**> 60 years**	541	< 0.001	2.040	1.567–2.655	< **0.001**	**2.186**	**1.665–2.871**
**Gender**							
Male	680	reference			reference		
**Female**	593	0.003	0.663	0.507–0.868	**0.003**	**0.659**	**0.502–0.864**
Race							
White	1022	reference					
Black	131	0.457	0.840	0.530–1.331			
Other	120	0.334	0.777	0.466–1.296			
**Site**							
Head	417	reference			reference		
**Body**	169	0.008	0.494	0.294–0.828	**0.008**	**0.492**	**0.290–0.833**
**Tail**	469	0.041	0.729	0.538–0.987	**0.031**	**0.710**	**0.519–0.970**
Other	218	0.163	0.768	0.530–1.113	0.180	0.768	0.522–1.130
Surgical Procedures							
Enucleation	20	reference					
PP	736	0.413	0.618	0.196–1.952			
TP	136	0.693	1.268	0.390–4.120			
Whipple	348	0.846	1.121	0.354–3.549			
Surgery NOS	33	0.341	0.483	0.108–2.161			
Tumor Size							
≤ 2 cm	347	reference					
> 2 cm	926	0.006	1.650	1.155–2.358			
Lymph Node Metastasis							
Negative	742	reference					
Positive	531	< 0.001	1.914	1.467–2.497			
Number of Examined Lymph Nodes							
≤ 12		reference					
> 12		0.074	1.276	0.977–1.667			
**SEER Grade**							
I	784	reference			reference		
II	185	0.491	1.166	0.754–1.802	0.623	1.118	0.718–1.740
**III**	95	**<** 0.001	5.073	3.516–7.321	**< 0.001**	**3.645**	**2.501–5.310**
**IV**	16	**<** 0.001	4.071	1.886–8.789	**0.008**	**2.876**	**1.320–6.270**
**Unknown**	193	0.001	1.752	1.259–2.438	**0.003**	**1.665**	**1.193–2.324**
**AJCC Stage 6th**							
I	493				reference		
II	513	0.001	1.906	1.319–2.754	0.128	1.411	0.906–2.197
**III**	24	0.001	3.457	1.620–7.376	**0.008**	**2.933**	**1.325–6.490**
**IV**	243	< 0.001	3.972	2.736–5.766	**< 0.001**	**3.297**	**2.089–5.203**
**Lymph Node Ratio**							
LNR = 0	742	reference			reference		
0 < LNR ≤ 0.40	369	0.001	1.665	1.233–2.249	0.621	1.095	0.765–1.567
**LNR > 0.40**	162	< 0.001	2.445	1.749–3.419	**0.012**	**1.650**	**1.117–2.438**

*Only factor with *P*-value ≤ 0.05 was included in multivariate analysis; variable and *P*-value in bold mean statistically significant in multivariate analysis. PP, Partial Pancreatectomy; TP, Total Pancreatectomy; NOS, not otherwise specified; SEER Grade [34]; AJCC, American Joint Committee on Cancer.

### The extent of examined lymph nodes and overall survival

The most appropriate cut-off value of ELNs for entire cohort and the patients without lymph node metastasis were 12 and 28, respectively. Surprisingly, we found that the extent of ELNs was not a significantly beneficial survival factor in either entire cohort (ELNs ≤ 12 vs. ELNs > 12, 92.363 months ± 1.978 months vs. 85.285 months ± 2.582 months, *P =* 0.072) (Figure 2B) or in patients without lymph node metastasis (ELNs ≤ 28 vs. ELNs > 28, 98.231 months ± 2.106 months vs. 72.246 months ± 9.734 months, *P =* 0.108) (Figure 2C).

### The lymph node ratio and overall survival

**Figure 3:**
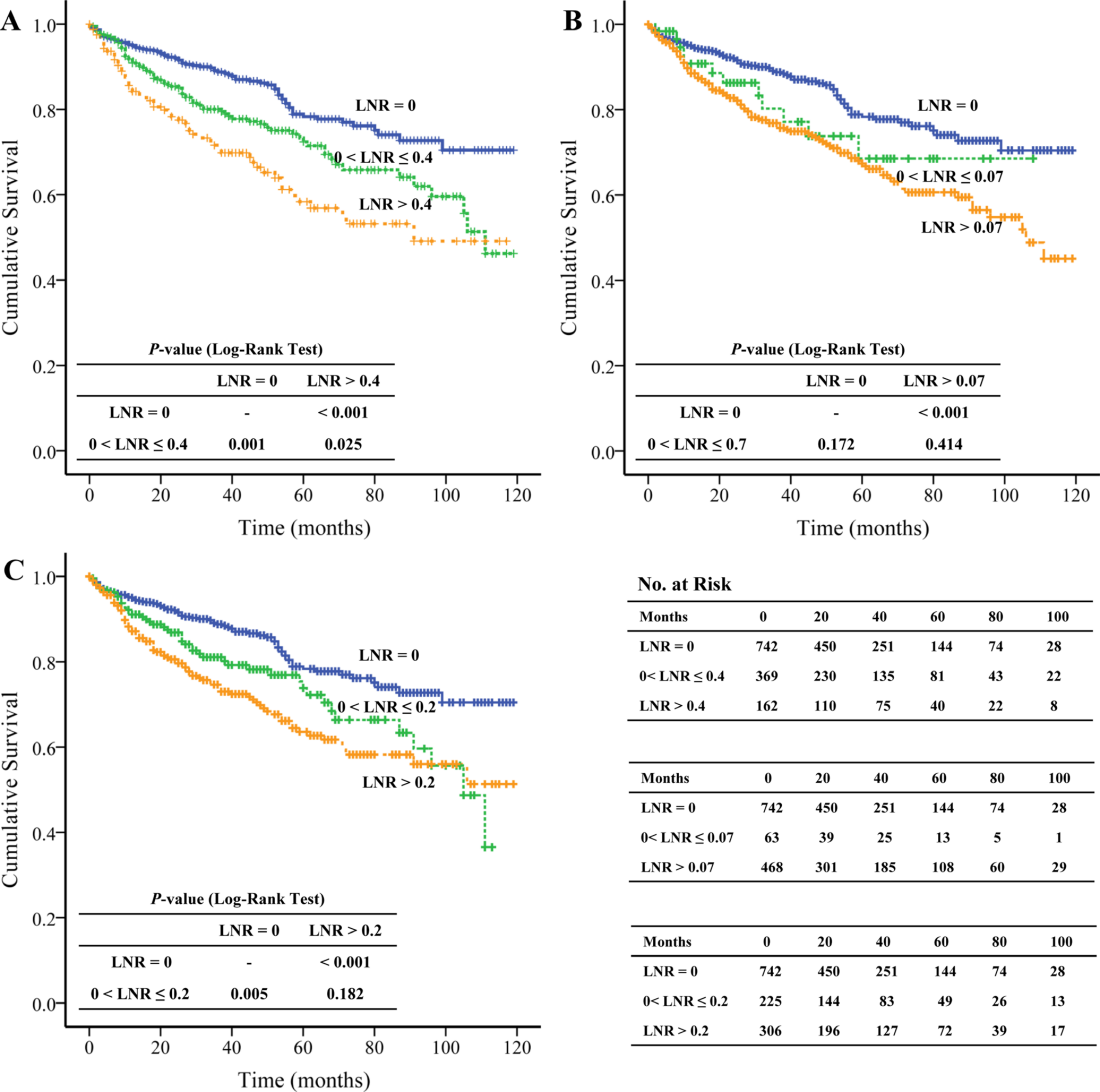
The Kaplan-Meier and log-rank tests of overall survival based on different cut-off values of lymph node ratio

The most appropriate cut-off value of LNR was 0.40 in our study; and we found that compared to the OS of patients with LNR = 0 (97.615 months ± 2.086 months) and 0 < LNR ≤ 0.40 (86.468 months ± 3.006 months), LNR > 0.40 exhibited significantly (*P* < 0.001; *P* = 0.025; respectively) shorter OS (75.473 months ± 4.287 months) (Figure 3A). The multivariate analysis showed that LNR > 0.40 was an independent prognostic factor (HR = 1.650, 95% CI: 1.117–2.438, *P* = 0.012) (Table 2).

We also evaluated the predictive role of LNR > 0.2 and LNR > 0.07 reported by Boninsegna et al. [19] and Ricci et al. [20], respectively. We found that compared to the OS of patients with LNR= 0 (97.615 months ± 2.086 months), patients with LNR > 0.2 and LNR > 0.07 presented significantly (both *P* < 0.001) shorter OS (81.004 months ± 3.257 months and 82.245 months ± 2.648 months, respectively) (Figure 3B and 3C). The univariate analysis demonstrated that LNR > 0.20 and LNR > 0.07 significantly (both *P* < 0.001) increased the risk of death (HR = 2.095, 95% CI: 1.561–2.812 and HR = 1.962, 95% CI: 1.496–2.574, respectively). However, the multivariate analysis failed to show that LNR > 0.20 and LNR > 0.07 were associated with OS (Supplementary Table 1).

### The independent prognostic factors and predictive power

Besides LNR > 0.40, the multivariate analysis also confirmed that age > 60 years (*P* < 0.001), advanced SEER grade (grade III, *P* < 0.001; grade IV, *P* = 0.008) and AJCC TNM staging (stage III, *P =* 0.008; stage IV, *P* < 0.001) were the adverse predict factors of OS in pNENs; and female (*P* = 0.003), tumor located in body (*P* = 0.008) or tail (*P* = 0.031) were the beneficial factors. This prognosis model (age, gender, tumor primary site, SEER grade, AJCC TNM staging and LNR) had acceptable discrimination (Harrell’s concordance index, 0.731, 95% CI: 0.689–0.773); and it was significantly better than SEER grade (Harrell’s concordance index, 0.636, 95% CI: 0.597–0.676) or AJCC TNM staging (Harrell’s concordance index, 0.636, 95% CI: 0.599–0.673).

### The LNR and clinicopathological characteristics

The Spearman’s rank correlation showed a high LNR was positive correlated with an advanced AJCC TNM staging (r_s_ = 0.604, *P* < 0.001). Similarly, the higher LNR showed positive correlation with bigger tumor size (r_s_ = 0.273, *P* < 0.001) and advanced SEER grade (r_s_ = 0.136, *P* < 0.001) (Table 3).

**Table 3:** Clinicopathological characters correlations with lymph node ratio (LNR)

Variable	LNR = 0	0 < LNR ≤ 0.4	LNR > 0.4	*P*-value	r_s_
	(*N* = 742)	(*N* = 369)	(*N* = 162)		
**Tumor Size**				**< 0.001^&^**	**0.273**
≤ 2 cm	278 (37.5%)	55 (14.9%)	14 (8.6%)		
> 2 cm	464 (62.5%)	314 (85.1%)	148 (91.4%)		
**SEER Grade**				**< 0.001^&^**	**0.136**
I	487 (65.6%)	219 (59.3%)	78 (48.1%)		
II	108 (14.6%)	58 (15.7%)	19 (11.7%)		
III	32 (4.3%)	41 (11.1%)	22 (13.6%)		
IV	6 (0.8%)	7 (1.9%)	3 (1.9%)		
**AJCC stage 6th**				**< 0.001^&^**	**0.604**
I	493 (66.4%)	0 (0%)	0 (0%)		
II	166 (22.4%)	251 (68.0%)	96 (59.3%)		
III	11 (1.5%)	9 (2.4%)	4 (2.5%)		
IV	72 (9.7%)	109 (29.5%)	62 (38.3%)		

SEER Grade [34]; AJCC, American Joint Committee on Cancer; ^&^Spearman’s rank correlation, r_s_ correlation coefficient; Variable and *P*-value in bold mean statistically significant.

## DISCUSSION

Currently, the AJCC and the European Neuroendocrine Tumor Society staging classification use the regional lymph node metastasis as a prognostic indicator of pNENs [21]. However, several studies proved contrary conclusions [22–25]. The conflicting results may be due to the incomplete lymphadenectomy or inadequate histopathological examination [26]. As the total number of ELNs rises, the number of metastatic lymph nodes also rises; and previous study demonstrated the number of ELNs were associated with the accuracy of lymph node staging and OS in PDAC, especially in patients without lymph node metastasis [15]. Thus, the International Study Group on Pancreatic Surgery recommended that at least 12 lymph nodes should be examined for PDAC [27].

In the present study, we only included patients underwent lymphadenectomy and the median of ELNs was 10 (IQR, 5–16). The most appropriate cut-off value of ELNs for entire cohort and pNENs without lymph node metastasis were 12 and 28, respectively. Surprisingly, we found the extent of ELNs failed to demonstrate any survival benefit in entire cohort (ELNs > 12 vs. ELNs ≤ 12) or in the negative lymph node metastasis pNENs (ELNs > 28 vs. ELNs ≤ 28). Similar to our findings, Conrad et al. [28] also demonstrated that the extent of ELNs (ELNs ≥ 10) failed to show significant survival advantage.

As mentioned by previous studies [7, 8], we also found the OS of pNENs with lymph node metastasis (82.965 months ± 2.504 months) was significantly (*P* < 0.001) shorter than that of pNENs without lymph node metastasis (97.615 months ± 2.093 months). Moreover, the lymph node metastasis significantly (*P* < 0.001) increased the risk of death (HR = 1.914; 95% CI: 1.467–2.497). However, the multivariate analysis failed to show lymph node metastasis was an independent prognostic factor in pNENs.

Recently, LNR has been demonstrated as a superior prognostic parameter than lymph node metastasis in PDAC [10, 11, 15], bladder [29], esophageal [30], and colon cancers [31]. Boninsegna et al. [19] and Ricci et al. [20] also demonstrated that LNR was an independent prognostic factor of recurrence in pNENs. However, Murakami et al. [8] reported LNR was not an independent prognostic factor of OS in pNENs.

We included 1,273 pathologically confirmed pNENs in our study and the most appropriate cut-off value of LNR was 0.40. The Kaplan-Meier and log-rank test demonstrated the OS of pNENs with LNR > 0.40 (75.473 months ± 4.287 months) was significantly shorter than that of pNENs with LNR = 0 (97.615 months ± 2.086 months*, P* < 0.001) and 0 < LNR ≤ 0.40 (86.468 months ± 3.006 months, *P* = 0.025), respectively; not only the univariate analysis but also the multivariate analysis showed LNR was an independent prognostic factor. To our knowledge, the present study is the largest population-based study to assess the OS benefit of LNR in pNENs.

Ricci et al. [20] and Boninsegna et al. [19] reported LNR > 0.07 and LNR > 0.20 were independent adverse predictors of recurrence, respectively. However, they did not discuss the relationship between LNR and OS. We found that compared to LNR = 0, LNR > 0.07 and LNR > 0.20 also significantly (both *P* < 0.001) increased the risk of death (HR = 1.962, 95% CI: 1.496–2.574; HR = 2.095, 95% CI: 1.561–2.812, respectively). However, the multivariate analysis failed to show LNR > 0.07 or LNR > 0.20 was an independent prognostic factor of OS. Our findings were consistent with those of Murakami et al. [8]. The authors reviewed the records of 119 consecutive patients with pancreatic ductal carcinoma and they also found LNR > 0.2 was not an independent prognostic factor of OS.

In contrast to pNENs, several studies had demonstrated LNR > 0.2 was an independent negative prognostic factor of OS in PDAC [32, 33]. The contradictory results can notably be explained by the relatively indolent physiological behavior of pNENs. pNENs are characterized by long term survival, even if lymph node metastases are present. Thus, the low cut-off value of LNR (0.20) may limit the identification of LNR in OS.

Besides LNR > 0.40, age older than 60 years, advanced AJCC stage, and SEER grade, the multivariate Cox regressions also demonstrated that male and primary tumor located in pancreatic head were associated with poor outcome. This is probably due to that the carcinoid syndrome was more frequently in female patients; and this increased the likelihood of diagnosis and surgical treatment in the early lesions for female patients [34]. Hashim et al. [35] reported that compared to pancreas body or tail, the head was more likely associated with lymph node metastasis; and this can explain the adverse prognostic role of primary tumor located in pancreas head.

We also found that the higher LNR was positively correlated with bigger tumor size, advanced AJCC stage, and SEER grade. To our knowledge, this is the first study to investigate the correlation between LNR and clinicopathological features.

Notably, there were several limitations to our study. First, due to the constraints of the SEER database, we failed to evaluate the role of Ki-67, mitotic index, lymphovascular invasion, resection margin in OS. Second, this was a retrospective study; thus, the selection bias was inevitable.

In summary, our study demonstrated that it was LNR, not the number of ELNs or lymph node metastasis, proved to be an independent prognostic indicator. In the future, it is better to take into account the LNR for the pNENs staging classification; and further prospective study is needed to determine these findings.

## MATERIALS AND METHODS

### Patient population

To identify the pancreatic tumor, the topography codes (C25 Pancreas, C25.0-C25.9) of International Classification of Diseases for Oncology, Third Edition (ICD-O-3) were used. Then the pNEN cases were retrieved based on the following morphology codes: 8013 large cell neuroendocrine carcinoma, 8041 small cell carcinoma, 8150 islet cell carcinoma, 8151 malignant beta cell tumor, 8152 malignant alpha cell tumor, 8153 malignant gastrinoma, 8155 VIPoma, 8156 somatostatin cell tumor, 8157 malignant enteroglucagonoma, 8240 carcinoid, 8241 argentaffin carcinoid tumor, 8242 enterochromaffin cell tumor, 8243 mucocarcinoid tumor, 8246 neuroendocrine carcinoma, and 8249 atypical carcinoid tumor.

### Inclusion and exclusion criteria

Only patients microscopically diagnosed as pNENs and underwent surgical resection were included. Cases without precise data for the following variables were excluded: race, tumor size, the number of lymph node metastasis or ELNs, and AJCC TNM staging. Large cell neuroendocrine carcinoma (ICD-O-3, 8013) and small cell carcinoma (ICD-O-3, 8041) mostly originating from lung were also excluded [3].

### Outcome and variables

The primary outcome was OS and the following variables were considered as potential prognostic factors of OS: age; gender (male and female); race (white, black. other: American Indian/AK Native, Asian/Pacific Islander and unknown); primary tumor site (head, body, tail, other: islets of Langerhans, other specified parts of pancreas, overlapping lesion of pancreas, not otherwise specified); surgical procedures (enucleation, partial pancreatectomy, total pancreatectomy, whipple, surgery not otherwise specified); tumor size; lymph node metastasis; ELNs; LNR; SEER grade and AJCC TNM staging (sixth edition).

Not all of the SEER registries reported tumor grade according to the WHO 2010 classification [36]. Therefore, SEER database used four tumor grades based on the basis of morphological description (ICD-O-3) in pathology report: SEER grade I including tumors classified as well differentiated; grade II including those classified as moderately differentiated; grade III including those classified as poorly differentiated, and grade IV including those classified as undifferentiated or anaplastic [34].

### The cut-off value of continuous variables

Age and tumor size were defined as two-category variable according to previous studies: age ≤ 60 years vs. age > 60 years [23]; tumor size ≤ 2 cm vs. tumor size > 2 cm [8]. The cut-off value of ELNs and LNR were determined according to the Youden’s index [37]; and the ELNs were also analyzed as two-categories. We hypothesized that the lymph node metastasis was a prognostic factor of OS. Thus, the LNR was analyzed in three categories (LNR = 0, LNR between 0 and cut-off value; LNR > cut-off value) and then it was also analyzed according to the cut-off value of 0.07 and 0.20 reported by Ricci et al. [20] and Boninsegna et al. [19], respectively.

### Data analysis and statistics

The continuous non-normal distribution variables were expressed as median and IQR, and the categorical or ordinal variables were presented as frequencies and proportions. The OS was analyzed using the Kaplan-Meier and log-rank tests and presented as mean ± standard deviation. Univariate (enter) and multivariate (forward stepwise regression) Cox proportional hazards models were used to identify the independent factors associated with OS. The Harrell’s concordance index was used to evaluate the combined predictive power of all independent prognostic factors [38]. If the value is more than 0.70, it can be concluded that the model has an acceptable discriminatory capability [39]. Spearman’s rank correlation coefficient, r_s_, was used to quantify the correlation between LNR and clinicopathological characteristics. r_s_ ranged from −1 to +1, where −1 indicates a perfect negative association of ranks, zero indicates no association between ranks and +1 refers to a perfect association of ranks. All statistical analyses were performed using SPSS version 19.0 (IBM Corporation. Armonk, NY, USA). *P*-value ≤ 0.05 was considered statistically significant.

## SUPPLEMENTARY MATERIALS TABLE





## Abbreviations

pNENs: pancreatic neuroendocrine neoplasms
PDAC: pancreatic ductal adenocarcinoma
LN: lymph node
ELNs: examined lymph nodes
LNR: lymph node ratio
SEER: Surveillance, Epidemiology, and End Results
OS: overall survival
AJCC TNM stage: American Joint Committee on Cancer Stage, Sixth Edition
