# Supplementary material for: Lymph node ratio, but not the total number of examined lymph nodes or lymph node metastasis, is a predictor of overall survival for pancreatic neuroendocrine neoplasms after surgical resection
Source: Oncotarget. 2017 Jul 12;8(51):89245–55. doi: 10.18632/oncotarget.19184 (PMC5687686; doi:10.18632/oncotarget.19184)
Supplement: Supplementary file 1 [file oncotarget-08-89245-s001.pdf]

Lymph node ratio, but not the total number of examined lymph nodes or lymph node metastasis, is a predictor of overall survival for pancreatic neuroendocrine neoplasms after surgical resection

SUPPLEMENTARY MATERIALS

Supplementary Table 1: Univariate and multivariate Cox regression analysis. See_Supplementary_Table_1
