# Supplementary material for: Lymph node ratio, but not the total number of examined lymph nodes or lymph node metastasis, is a predictor of overall survival for pancreatic neuroendocrine neoplasms after surgical resection
Source: Oncotarget. 2017 Jul 12;8(51):89245–55. doi: 10.18632/oncotarget.19184 (PMC5687686; doi:10.18632/oncotarget.19184)
Supplement: Supplementary file 2 [file oncotarget-08-89245-s002.docx]

Supplementary Table 1. Univariate and multivariate Cox regression analysis

| Variable | N | Univariate | | | Multivariate (Model 1 and Model 2)^$^ | | |
| --- | --- | --- | --- | --- | --- | --- | --- |
|  |  | *P*-value* | HR | 95%CI | *P*-value | HR | 95%CI |
| **Age** |  |  |  |  |  |  |  |
| ≤60 years | 732 | reference | | | reference | | |
| **>60 years** | 541 | <0.001 | 2.040 | 1.567-2.655 | **< 0.001** | **2.183** | **1.662-2.867** |
| **Gender** |  |  |  |  |  |  |  |
| Male | 680 | reference | | | reference | | |
| **Female** | 593 | 0.003 | 0.663 | 0.507-0.868 | **0.005** | **0.677** | **0.517-0.887** |
| Race |  |  |  |  |  |  |  |
| White | 1022 | reference | | |  |  |  |
| Black | 131 | 0.457 | 0.840 | 0.530-1.331 |  |  |  |
| Other | 120 | 0.334 | 0.777 | 0.466-1.296 |  |  |  |
| **Site** |  |  |  |  |  |  |  |
| Head | 417 | reference | | | reference | | |
| **Body** | 169 | 0.008 | 0.494 | 0.294-0.828 | **0.009** | **0.497** | **0.293-0.843** |
| **Tail** | 469 | 0.041 | 0.729 | 0.538-0.987 | **0.043** | **0.726** | **0.533-0.991** |
| Other | 218 | 0.163 | 0.768 | 0.530-1.113 | 0.199 | 0.778 | 0.530-1.142 |
| Surgical Procedures | | | |  |  |  |  |
| Enucleation | 20 | reference | | |  |  |  |
| PP | 736 | 0.413 | 0.618 | 0.196-1.952 |  |  |  |
| TP | 136 | 0.693 | 1.268 | 0.390-4.120 |  |  |  |
| Whipple | 348 | 0.846 | 1.121 | 0.354-3.549 |  |  |  |
| Surgery NOS | 33 | 0.341 | 0.483 | 0.108-2.161 |  |  |  |
| Tumor Size |  |  |  |  |  |  |  |
| ≤ 2 cm | 347 | reference | | |  |  | |
| > 2 cm | 926 | 0.006 | 1.650 | 1.155-2.358 |  |  |  |
| Lymph Node Metastasis | | | | | | | |
| Negative | 742 | reference | | |  |  | |
| Positive | 531 | < 0.001 | 1.914 | 1.467-2.497 |  |  |  |
| Number of Examined Lymph Nodes | | | |  |  |  |  |
| ≤12 | 795 | reference | | |  |  |  |
| > 12 | 478 | 0.074 | 1.276 | 0.977-1.667 |  |  |  |
| **SEER Grade** |  |  |  |  |  |  |  |
| I | 784 | reference | | | reference | | |
| II | 185 | 0.491 | 1.166 | 0.754-1.802 | 0.678 | 1.098 | 0.706-1.707 |
| **III** | 95 | ＜0.001 | 5.073 | 3.516-7.321 | **＜0.001** | **3.626** | **2.491-5.280** |
| **IV** | 16 | ＜0.001 | 4.071 | 1.886-8.789 | **0.014** | **2.633** | **1.213-5.717** |
| **Unknown** | 193 | 0.001 | 1.752 | 1.259-2.438 | **0.001** | **1.735** | **1.246-2.416** |
| **AJCC Stage 6^th^** |  |  |  |  |  |  |  |
| I | 493 |  |  | | reference | | |
| **II** | 513 | 0.001 | 1.906 | 1.319-2.754 | 0.007 | 1.676 | 0.149-2.446 |
| **III** | 24 | 0.001 | 3.457 | 1.620-7.376 | **0.003** | **3.284** | **1.503-7.175** |
| **IV** | 243 | < 0.001 | 3.972 | 2.736-5.766 | **<0.001** | **3.955** | **2.701-5.789** |
| Lymph Node Ratio 1 | | | | | | | |
| LNR = 0 | 742 | reference | | |  | | |
| 0< LNR≤0.07 | 63 | 0.166 | 1.530 | 0.838-2.794 |  |  |  |
| LNR >0.07 | 468 | < 0.001 | 1.962 | 1.496-2.574 |  |  |  |
| Lymph Node Ratio 2 | | | | | | | |
| LNR = 0 | 742 | reference | | |  |  |  |
| 0< LNR≤0.20 | 225 | 0.005 | 1.655 | 1.166-2.347 |  |  |  |
| LNR >0.20 | 306 | < 0.001 | 2.095 | 1.561-2.812 |  |  |  |

*only factor with *P*-value ≤0.05 was included in multivariate analysis; Variable and *P*-value in bold mean statistically significant in multivariate analysis; ^$^ factors in model 1: age, gender, site, tumor size, lymph node metastasis, SEER grade, AJCC stage 6th, lymph node ratio 1; factors in model 2: age, gender, site, tumor size, lymph node metastasis, SEER grade, AJCC stage 6^th^, lymph node ratio 2; and both models demonstrated only age, gender, site, SEER grade and AJCC stage were the independent prognostic factors.
